# NT-proBNP Reflects Left Ventricular Hypertrophy Rather than Left Ventricular Dilatation or Systolic Dysfunction in Patients with Fabry Disease

**DOI:** 10.3390/jcm13195953

**Published:** 2024-10-07

**Authors:** Constantin Gatterer, Dietrich Beitzke, Gere Sunder-Plassmann, Maximilian Friedl, Philipp Hohensinner, Christopher Mann, Markus Ponleitner, Senta Graf, Max Lenz

**Affiliations:** 1 Division of Cardiology, Department of Internal Medicine II, Medical University of Vienna, 1090 Vienna, Austria; constantin.gatterer@meduniwien.ac.at (C.G.); senta.graf@meduniwien.ac.at (S.G.); 2 Department of Biomedical Imaging and Image-Guided Therapy, Medical University of Vienna, 1090 Vienna, Austria; 3 Division of Nephrology and Dialysis, Department of Internal Medicine III, Medical University of Vienna, 1090 Vienna, Austria; 4 Department of Neurology, Medical University of Vienna, 1090 Vienna, Austria

**Keywords:** Fabry disease, cardiomyopathy, lysosomal storage disease, biomarker, NT-proBNP, cardiac MRI, echocardiography

## Abstract

**Background**: The diagnosis and follow-up of cardiac involvement in Fabry disease constitutes an important challenge for clinicians caring for affected patients. Combining cardiac imaging with laboratory biomarkers appears most appropriate for longitudinal monitoring. Therefore, we examined the use of NT-proBNP and its association with imaging findings in patients with Fabry disease. **Methods**: We analysed cardiac MRI and echocardiography data, as well as laboratory results, from a single-centre prospective registry. **Results**: Repetitive follow-ups of 38 patients with Fabry disease, of whom 18 presented with left ventricular hypertrophy (LVH), revealed a correlation of NT-proBNP with left ventricular (LV) interventricular septal thickness, LV maximum wall thickness, LV and right ventricular (RV) mass index and trabecular mass in patients with LVH. Patients without LVH did not exhibit any tangible association between NT-proBNP and the mentioned parameters. Conversely, we could not detect an association of NT-proBNP with impairment of LV or RV ejection fraction or diastolic volume. **Conclusions**: NT-proBNP plays a pivotal role as a biomarker for cardiac involvement in patients with Fabry disease. Interestingly, in this specific population with mostly preserved ejection fraction, it seems to reflect ventricular hypertrophy rather than ventricular dysfunction or dilatation. While strong associations were found in hypertrophic patients, NT-proBNP’s prognostic value appears limited in non- or pre-hypertrophic stages.

## 1. Introduction

Fabry disease (Online Mendelian Inheritance in Man [OMIM] #300644) is an X-linked lysosomal storage disorder caused by deficient activity of the enzyme alpha-galactosidase A and results in the accumulation of glycosphingolipids within the cells of various organ systems [1]. Besides renal, neurological, gastrointestinal, ophthalmological and dermatological manifestations, cardiac involvement in Fabry disease is common. It is referred to as Fabry cardiomyopathy and occurs in up to 50% of the female and 60% of the affected male patients [2]. Moreover, cardiovascular complications remain a leading cause of morbidity and mortality in Fabry patients. Progressive glycosphingolipid deposition and subsequent processes within cardiomyocytes and cells of the cardiac conduction system play a pivotal role in the pathomechanism of the disease. The resulting effects range from left ventricular wall thickening, diastolic dysfunction, arrhythmia, and valvular dysfunction over myocardial fibrosis to systolic dysfunction in advanced stages [3,4]. However, disease progression is challenging to predict, as it varies between individual patients despite them carrying the same genetic variant [5]. Therefore, regular evaluations of the typically affected organ systems, including the kidneys, the central nervous system and the heart, are recommended [6,7,8,9,10,11].

Besides imaging techniques such as cardiac magnetic resonance imaging (CMR) and transthoracic echocardiography (TTE), the assessment of cardiac laboratory biomarkers appears useful. In the literature, N-terminal pro-brain natriuretic peptide (NT-proBNP) and troponins are proposed to be beneficial for the early detection of myocardial involvement [12]. Yet, the use of NT-proBNP as a biomarker remains largely underexplored, and data evaluating its long-term progression and detailed associations with imaging parameters are missing so far. This study aims to shed some light on repetitive NT-proBNP measurements in addition to comprehensive cardiac imaging in a real-life cohort of patients with Fabry disease. In this regard, we aim to provide a detailed understanding of NT-proBNP and its role as a biomarker in different stages of the disease. Furthermore, Fabry-specific findings, such as associations with globotriaosylsphingosine (LysoGb3) and low myocardial T1, representing myocardial glycosphingolipid accumulation, are to be investigated.

## 2. Materials and Methods

### 2.1. Patient Inclusion and Follow-Ups

The present study comprises patients enrolled in the prospective registry “KarMA”. All individuals were consecutively included from October 2011 to April 2019. Patients gave written informed consent and were stratified according to their genetic variants and the requirement of specific therapy (ERT or chaperone therapy). All individuals were 18 years of age or older at the time of their inclusion. Patients with or without indication for specific therapy were included to reflect the heterogenous course of disease. Follow-ups were scheduled based on the severity of the disease, either annually or at longer intervals. The follow-ups were clustered into 30 (±15), 60 (±15) and 90 (±15) months to assess the longitudinal development of NT-proBNP in patients with or without specific therapy. If multiple visits were available in a selected time frame (for example, due to unscheduled visits), the visit closest to the defined follow-up (30, 60 or 90 months) was included in the final analysis. Correlations and group comparisons were calculated using all available data and included all follow-ups unless indicated otherwise. This study was approved by the institutional ethics advisory committee of the Medical University of Vienna (IRB Nr.: 1867/2021) and complies with the declaration of Helsinki and all of its further amendments.

### 2.2. Data Assessment and Blood Sampling

Baseline characteristics, patient history, concomitant medication, therapy status and markers of Fabry-associated organ manifestation were recorded during each visit. Particularly, cardiac and renal function were evaluated through analyses of respective biomarkers and imaging, whereas symptoms were evaluated by a structured anamnestic interview. Moreover, standard laboratory values were measured via the central laboratory of the Vienna General Hospital according to local standards and techniques. A standard immunoassay was performed for NT-proBNP (NT-proBNPII, Cobas/Roche, Basel, Switzerland). In addition, one serum separator tube, one ethylenediaminetetraacetic acid (EDTA) tube and one 3.8% sodium citrate vacuette tube (all Greiner Bio-One, Kremsmünster, Austria) were collected. The blood was consecutively centrifuged with 3000 RPM at 4 °C and stored in a biobank at −80 °C for potential later analyses. A reference value ≤ 125 pg/mL was considered for NT-proBNP. Disease severity was measured in patients with a complete interdisciplinary work-up during the most recent follow-up using the Fabry disease-specific score Fabry outcome study—Mainz severity score index (FOS-MSSI). As the FOS-MSSI assesses the different types of organ involvement in Fabry disease, it is used as a surrogate for disease severity and progression [13].

### 2.3. LysoGb3 Measurements

EDTA blood samples were used to prepare Dried Blood Spot (DBS) cards (ARCHIMED Life Science GmbH, Vienna, Austria and Centogene GmbH, Rostock, Germany), which were mailed to the respective laboratories. Mass spectrometry was utilised to measure LysoGb3 concentration as described previously [14,15]. A reference value of <3.5 ng/mL was considered for ARCHIMED Life Science, and a reference value of ≤1.8 ng/mL for Centogene DBS cards.

### 2.4. Cardiac Imaging

TTE and CMR were carried out during the scheduled visits. CMR 1.5 tesla examinations were performed on a Siemens Avanto Fit MRI scanner (Siemens Healthineers AG, Erlangen, Germany) with a standardised multiparametric protocol [16]. Image analysis was performed utilising post-processing software (Medis Suite MR 4.0.70.4, Medis Medical Imaging, Leiden, The Netherlands). Left ventricular (LV) and right ventricular (RV) dimensions and function were calculated from the 1.5T cine-balanced steady-state free precession short-axis images taken during end-diastole and end-systole. Left atrial volume was calculated from 4-chamber and 2-chamber cine images using biplane tissue tracking of the endocardial border as described previously [17]. T1 measurement was performed in the mid-cavity septal region where values below 950 ms were defined as low T1 [18]. For the estimation of late gadolinium enhancement (LGE), 0.15 mL/kg bodyweight gadobutrol (Gadovist, Bayer, Berlin, Germany) was injected. The extent of LGE was calculated as a percentage of the left ventricular mass using the full width at half-maximum method. TTE (2D and Doppler) examinations were performed at the echocardiography laboratory at the Vienna General Hospital. Ultrasound systems with 3.5 MHz transducer (Vivid E9, Vivid S70, General Electric Healthcare, Chicago, USA) were used. Standard protocols were followed in accordance with the recommendations of the American Society of Echocardiography and the European Association of Cardiovascular Imaging [19]. Left ventricular function was visually estimated. Left ventricular wall thickening, referred to as left ventricular hypertrophy (LVH), was defined as an interventricular septum >12 mm (TTE) or LVMI, without papillary muscles, of >75 g/m^2^ for males and >59 g/m^2^ for females (CMR) [20].

### 2.5. Statistical Analysis

Categorical variables are presented as counts and percentages. They were analysed using Chi-square or Fisher’s exact test as applicable. Continuous variables are presented as median and interquartile ranges (IQR) and were tested for equality of variances using Levene’s test. The Kolmogorov–Smirnov test was used to differentiate between parametric and non-parametric data. Parametric values were analysed using the unpaired Student’s t-test, whereas non-parametric data were analysed using the Mann–Whitney U test. To compare multiple groups, one-way ANOVA with Bonferroni correction was utilised. For correlations of imaging parameters and NT-proBNP, the Spearman correlation coefficient (r) was applied. To further explore the prognostic value of NT-proBNP on the course of Fabry disease in individual patients, assessed by the FOS-MSSI, logistic regression was calculated. SPSS 26.0 (IBM Corporation, Armonk, NY, USA) was used for statistical interpretation. The significance level was set at < 0.05 for all tests and analyses unless indicated otherwise.

## 3. Results

### 3.1. Baseline Characteristics

A total of 38 patients and 94 visits (including the baseline) were analysed. Baseline characteristics can be found in Table 1 (stratified female vs. male patients) and Table 2 (stratified non-hypertrophic vs. hypertrophic patients). The median age of the depicted population was 41 years (IQR 27–50), and 65.8% of the patients were female. Moreover, 47.4% of the analysed individuals exhibited left ventricular hypertrophy, and 39.5% received specific therapy (31.6% enzyme replacement therapy and 7.9% chaperone). At baseline, there were no significant differences between male and female patients for NT-proBNP *(p* = 0.234) and the estimated glomerular filtration rate (*p* = 0.724). However, female patients exhibited significantly lower creatinine levels (*p* = 0.001) and albumin-to-creatinine ratios (*p* = 0.003). Comparing hypertrophic and non-hypertrophic patients, significant differences were found in LVMI (*p* = 0.001), LVEDV (*p* = 0.003) and septal T1 (*p* = 0.039).

### 3.2. Association of NT-proBNP, Specific Therapy and Left Ventricular Hypertrophy

**Figure 1:**
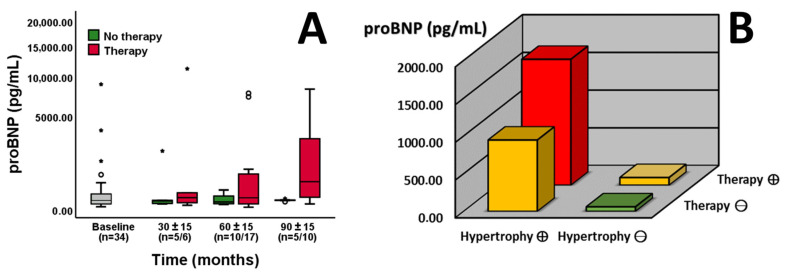
Longitudinal development of NT-proBNP values in patients with (red boxplots) or without (green boxplots) specific therapy ((**A**), *n* = indicated). There is a trend towards differences after 60 months (*p* = 0.083) and significant differences after 90 months (*p* = 0.020). NT-proBNP values of all available time points are stratified by therapy and the presence of left ventricular hypertrophy ((**B**), *n* = 87, the red bar indicates both factors, the yellow bar one, and the green bar none). Patients on specific therapy with left ventricular hypertrophy (red bar) display increased NT-proBNP levels in contrast to those without these two factors (green bar, *p* = 0.026). The Y-axis is shown as a percentage function (**A**) to highlight low NT-proBNP values. A *p*-value of <0.05 was considered statistically significant. “*” indicates outliers within the cohort.

**Figure 2:**
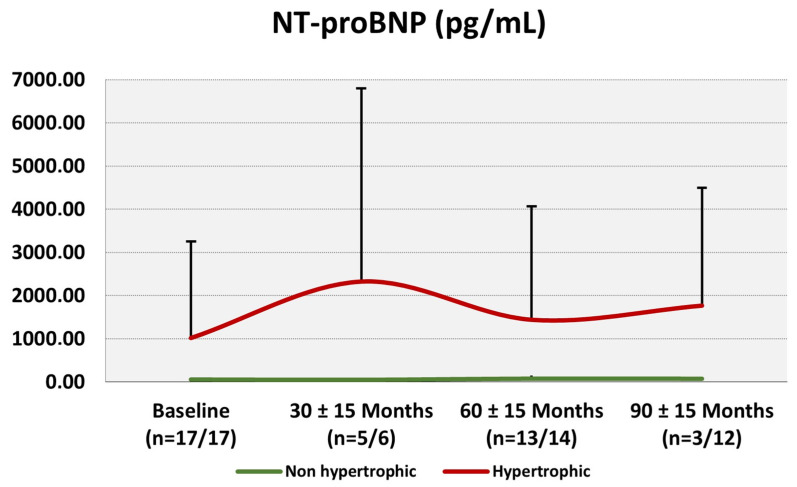
Changes in average (+standard deviation) NT-proBNP values from baseline to 30 ± 15, 60 ± 15 and 90 ± 15 months follow-ups. The red line represents hypertrophic individuals (defined as an interventricular septum >12 mm (TTE) or LVMI of >75 g g/m^2^ ♂ or >59 g g/m^2^ ♀), whereas the green line depicts non-hypertrophic patients.

Patient follow-ups were clustered into 30 (±15), 60 (±15) and 90 (±15) months to assess the longitudinal development of NT-proBNP in patients with or without specific therapy (enzyme replacement therapy or chaperone therapy). Comparing baseline and later visits, patients without specific therapy exhibited no significant changes in NT-proBNP levels (*p* = 0.769, Figure 1A). Comparing patients with and without therapy, a trend towards higher NT-proBNP values was detectable after 60 months in patients on therapy (*p* = 0.083, Figure 1A). Furthermore, significant differences between the two groups were present after 90 months (*p* = 0.020, Figure 1A). In addition, the patients’ NT-proBNP measurements were stratified based on therapy and the presence of left ventricular hypertrophy. Comparison of the resulting four groups highlighted significant differences between them (ANOVA: *p* = 0.022, Figure 1B). Particularly, patients on specific therapy with left ventricular hypertrophy exhibited increased NT-proBNP levels in contrast to those without these two factors (ANOVA: *p* = 0.026, Figure 1B). Changes in NT-proBNP levels are illustrated in hypertrophic and non-hypertrophic individuals according to follow-ups (Figure 2).

### 3.3. NT-proBNP and Echocardiography

To assess the association of NT-proBNP levels and echocardiographic measurements, correlations were calculated (shown in Table 3). Patients with left ventricular hypertrophy exhibited significant correlations with interventricular septal thickness (r = 0.678; *p* < 0.001), right atrial diameter (r = 0.684; *p* < 0.001), aorta ascendens diameter (r = 0.338; *p* = 0.031) and systolic pulmonary pressure (sPAP, r = 0.865; *p* < 0.001). Interestingly, these findings were only present in patients exhibiting left ventricular hypertrophy as compared to those without.

### 3.4. NT-proBNP and Cardiac Magnetic Resonance Imaging

Further correlations were calculated for NT-proBNP levels and CMR (shown in Table 4). Patients with left ventricular hypertrophy exhibited significant correlations with left ventricular myocardial mass (r = 0.538; *p* < 0.001), left ventricular mass index (r = 0.549; *p* < 0.001), left ventricular trabecular mass (0.526; *p* < 0.001), left ventricular maximum wall thickness (r = 0.717; *p* < 0.001), right ventricular myocardial mass (0.470; *p* = 0.003), right ventricular mass index (r = 0.428; *p* = 0.007) and right ventricular trabecular mass (r = 0.606; *p* = 0.007). Again, these findings were only observed in patients with left ventricular hypertrophy, not in those without.

### 3.5. NT-proBNP Reflects Hypertrophy Rather than Dilatation or Dysfunction

**Figure 3:**
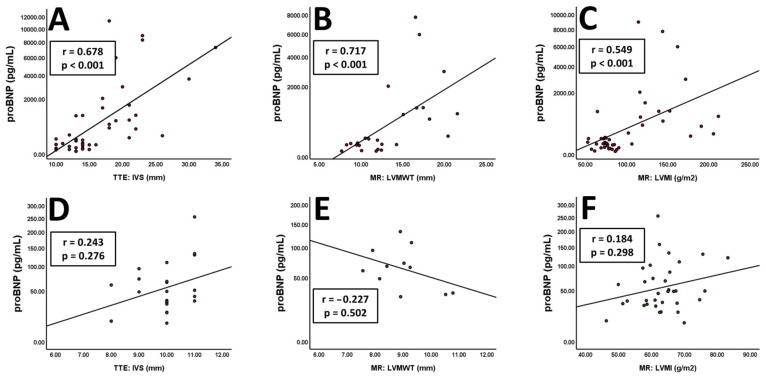
Correlations of NT-proBNP values and imaging markers of hypertrophy in patients with ((**A**) *n* = 47, (**B**) *n* = 27, (**C**) *n* = 46) or without ((**D**) *n* = 21, (**E**) *n* = 11, (**F**) *n* = 33) left ventricular hypertrophy. The Y-axis is shown as a percentage function to highlight low NT-proBNP values. A *p*-value of <0.05 was considered statistically significant.

Highly significant correlations were found for interventricular septal thickness (LVH, r = 0.678; *p* < 0.001 vs. no LVH, r = 0.243; *p* = 0.276, Figure 3A,D), left ventricular maximum wall thickness (LVH, r = 0.717; *p* < 0.001 vs. no LVH, r = −0.227; *p* = 0.502, Figure 3B,E) and left ventricular mass index (LVH, r = 0.549; *p* < 0.001 vs. no LVH, r = 0.184; *p* = 0.298, Figure 3C,F). In contrast to these hypertrophy-associated findings, no significant correlations were deducible for NT-proBNP and imaging markers reflecting ventricular dilatation or systolic dysfunction. These findings remained true in both hypertrophic and non-hypertrophic patients and are summarised in Supplementary Figure S4. Additionally, correlations in the total patient cohort resemble those with left ventricular hypertrophy (Supplementary Figure S1).

### 3.6. Associations of NT-proBNP and LysoGb3

**Figure 4:**
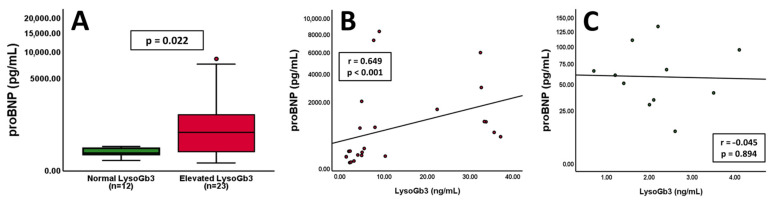
Patients with elevated LysoGb3 values exhibit increased NT-proBNP levels ((**A**), *p* = 0.022, *n* = indicated). Correlations of NT-proBNP and LysoGb3 values in patients with ((**B**), *n* = 24) or without ((**C**), *n* = 11) left ventricular hypertrophy. The Y-axis is shown as a percentage function to highlight low NT-proBNP values. A *p*-value of < 0.05 was considered statistically significant.

Associations of NT-proBNP and LysoGb3 are depicted in Figure 4. Patients with elevated LysoGb3 values exhibited increased levels of NT-proBNP compared to patients within the standard range (Figure 4A, *p* = 0.022). Additionally, a strong correlation between NT-proBNP and LysoGb3 was found in patients with left ventricular hypertrophy (Figure 4B, r = 0.649; *p* < 0.001). In contrast, individuals without left ventricular hypertrophy displayed no tangible correlation between the two markers (Figure 4C, r = −0.045; *p* = 0.894).

### 3.7. NT-proBNP, Low T1 and Diastolic Dysfunction

**Figure 5:**
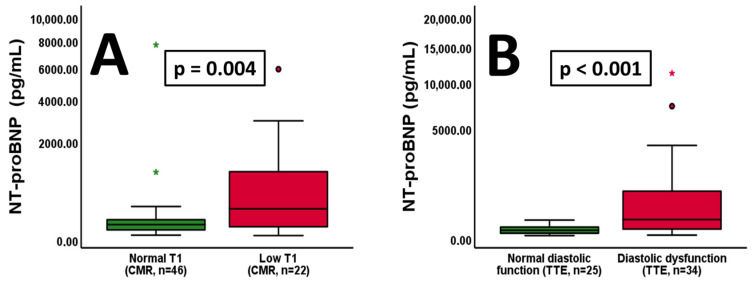
Patients with low T1 values exhibit increased NT-proBNP levels ((**A**), *p* = 0.004, *n* = indicated). Furthermore, individuals with left ventricular diastolic dysfunction show elevated NT-proBNP levels compared to those with normal function ((**B**), *p* < 0.001, *n* = indicated). The Y-axis is shown as a percentage function to highlight low NT-proBNP values. A *p*-value of <0.05 was considered statistically significant. “*” indicates outliers within the cohort.

In our cohort, patients with low T1 exhibited significantly increased levels of NT-proBNP compared to patients with normal T1 values (Figure 5A, *p* = 0.004). Moreover, individuals with diastolic dysfunction showed higher NT-proBNP values compared to those with normal function (Figure 5B, *p* < 0.001). In this context, left ventricular diastolic function may be interpreted in conjunction with left ventricular hypertrophy. In the total cohort, left ventricular systolic function did not vary between patients with or without hypertrophy (Supplementary Figure S2). However, patients exhibiting left ventricular hypertrophy showed significantly increased occurrence of left ventricular diastolic dysfunction (Supplementary Figure S2).

### 3.8. NT-proBNP and Right Ventricular Parameters

**Figure 6:**
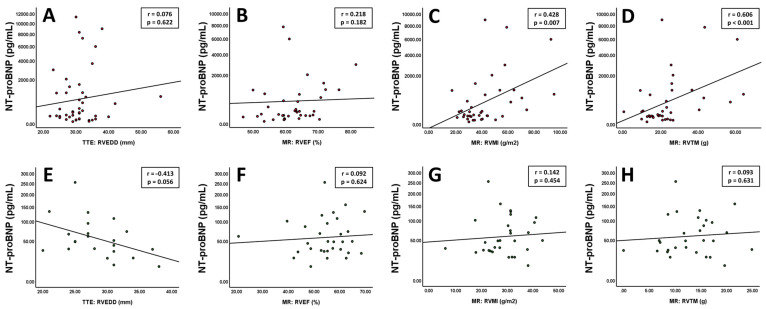
Patients with left ventricular hypertrophy show no correlations between NT-proBNP and right ventricular end-diastolic diameter ((**A**), *n* = 44) and right ventricular function ((**B**), *n* = 38) but with right ventricular mass index ((**C**), *n* = 38) and right ventricular trabecular mass ((**D**), *n* = 38). Individuals without hypertrophy exhibit no significant correlations with the mentioned parameters ((**E**) *n* = 22, (**F**) *n* = 31, (**G**) *n* = 31, (**H**) *n* = 29). The Y-axis is shown as a percentage function to highlight low NT-proBNP values. A *p*-value of <0.05 was considered statistically significant.

After focusing primarily on the left ventricle, key findings were validated for right ventricular parameters. Imaging markers of right ventricular function, dilatation and hypertrophy are displayed in Figure 6. In patients with left ventricular hypertrophy, no correlations were found for NT-proBNP and right ventricular end-diastolic diameter (Figure 6A, r = 0.076; *p* = 0.622) and right ventricular function (Figure 6B, r = 0.218; *p* = 0.182). In contrast, the right ventricular mass index (Figure 6C, r = 0.428; *p* = 0.007) and right ventricular trabecular mass (Figure 6D, r = 0.606; *p* < 0.001) exhibited significant correlations with NT-proBNP. In individuals without hypertrophy, none of the mentioned parameters showed any tangible correlations (Figure 6E,F).

### 3.9. NT-proBNP and Overall Disease Severity

Logistic regression revealed a significant influence of baseline NT-proBNP levels on the latest available complete FOS-MSSI Score (*n* = 26, *p* = 0.004; β = 0.57) after a mean follow-up period of 7.48 ± 2.58 years. However, this finding was only reproducible in the sub-group of patients with LVH (*p* = 0.031; β = 0.598) but not in those without (*p* = 0.162; β = 0.453).

## 4. Discussion

NT-proBNP has repeatedly been proven to be one of the most important prognostic biomarkers in patients with cardiomyopathies and heart failure with reduced as well as preserved ejection fraction [22,23]. Stimulated by cardiac wall stress due to myocardial dysfunction or dilatation, release and consecutive elevation of NT-proBNP in the sera of affected patients has become a cornerstone in managing heart failure, as it reflects ventricular volume overload and increased filling pressures [24,25]. More recently, it has been recognised as a valuable marker in the monitoring of Fabry disease patients. However, these findings are based on a small number of studies that assessed partial aspects of its relationship with cardiac manifestations [12]. While several other conditions, such as valvular pathologies, a variety of other cardiomyopathies, arrhythmia, pulmonary hypertension and renal dysfunction, may also lead to increased levels of NT-proBNP, the underlying mechanisms in Fabry disease appear unique [26]. Cardiomyocyte hypertrophy with consecutive myocardial wall thickening and diastolic dysfunction is caused by glycosphingolipid accumulation and subsequent processes such as inflammation, autophagy and mitochondrial dysfunction, as well as oxidative stress. These processes ultimately lead to apoptosis, necrosis and fibrosis and differentiate them from other causes of severe left ventricular hypertrophy, such as hypertrophic cardiomyopathy, in which sarcomeric dysfunction triggers signalling pathways leading to cardiomyocyte hypertrophy, cardiomyocyte disarray and fibrosis [4,27]. As left ventricular dilatation and the impairment of left ventricular systolic function due to replacement fibrosis only appear in very advanced stages, we assume that myocardial wall thickening and diastolic dysfunction are the main contributors to cardiac wall stress and elevations of NT-proBNP in patients with mild or moderate cardiac involvement of Fabry disease. Furthermore, renal involvement leading to chronic kidney disease is common in Fabry disease and could potentially impair the clearance of NT-proBNP. Arrhythmias, with atrial fibrillation being one of the most common, are also frequently found and could further contribute to the levels of NT-proBNP in affected patients due to atrial enlargement [4].

To the best of our knowledge, our study is the first to extensively investigate NT-proBNP in association with multimodal cardiac imaging parameters. Analysing the data from comprehensive CMR and TTE examinations, we identified a strong interdependency with markers of ventricular hypertrophy and diastolic dysfunction. In contrast, no compelling associations were found for ventricular dilatation or systolic dysfunction parameters. Additionally, a strong correlation between low septal T1 measurements and NT-proBNP was revealed. This might reflect the involvement of myocardial glycosphingolipid accumulation in the context of subsequently emerging hypertrophy and associated elevation of the biomarker.

Torralba-Cabeza et al. were the first to examine the use of NT-proBNP in patients with Fabry disease [28]. In 2011, the authors proved the biomarker to reflect organ involvement, as assessed by the Mainz severity score index and diastolic dysfunction. Later, the group of Coats et al. highlighted the role of NT-proBNP in combination with various other echocardiographic parameters. They found left atrial size and E/Ea ratio, reflecting the LV filling pressure, to be independently associated with the levels of NT-proBNP. However, they provided data that markers of LVH, including the left ventricular mass index, failed to show significant associations in the multivariate analysis [29]. In line with these previous studies, patients from our cohort who presented with diastolic dysfunction exhibited elevated levels of NT-proBNP. Furthermore, TTE-derived right atrial diameter and systolic pulmonary artery pressure correlated significantly with levels of the biomarker. Conversely, we found a weak correlation between the left ventricular ejection fraction and levels of NT-proBNP in the general cohort but not in the respective sub-groups with or without LVH.

In Fabry patients, our data suggest LVH to be the primary factor contributing to elevated NT-proBNP levels. Moreover, left ventricular systolic dysfunction or dilation appear less determining in our cohort. This hypothesis is supported by strong correlations between the biomarker and echocardiographically as well as CMR-measured parameters reflecting hypertrophy. In particular, interventricular septal thickness (TTE), maximum wall thickness (CMR), left ventricular myocardial mass (CMR), LVMI (CMR) and trabecular mass (CMR) were strongly associated with NT-proBNP. On the other hand, right and left ventricular end-diastolic diameters (TTE) or volumes (CMR) exhibited no tangible correlations. These findings appear interesting as NT-proBNP reflects left ventricular function and heart failure occurrence rather well in non-Fabry patients. Earlier reported elevations of NT-proBNP in patients with diastolic dysfunction are possibly intertwined with these hypertrophy-associated findings. A significant overlap of patients with diastolic dysfunction and LVH in our study highlights this possible relationship. While NT-proBNP was repeatedly proven to be a robust predictive marker for diastolic dysfunction in non-Fabry-disease patients, several previous studies also reported the interaction of NT-proBNP and left ventricular hypertrophy [24,30,31,32,33]. 

Among others, Linhart et al. discussed the frequent finding of aortic root dilatation in parallel or secondary to LVH as part of the complex of Fabry cardiomyopathy [34]. Interestingly, an increase in the ascending aorta diameter, particularly in patients with LVH, was also found to be associated with elevated NT-proBNP levels in our study.

Right ventricular involvement in Fabry disease has been reported previously. The findings range from ventricular hypertrophy and impaired ventricular free wall strain to impaired systolic function [35,36,37,38]. Accordingly, we found that NT-proBNP levels correlate well with right ventricular myocardial and trabecular mass. However, the evaluation of right ventricular dimensions and function remains challenging. Moreover, cardiac events and prognosis are thought to be mainly impacted by left-sided cardiac involvement [36]. Observations in our cohort are in line with this notion, as correlations with right ventricular parameters were only present in patients with left ventricular hypertrophy. Besides LVH, the decrease of myocardial T1 relaxation times, caused by glycosphingolipid accumulation and the presence of basal inferolateral LGE, reflecting myocardial fibrosis, are hallmarks of Fabry cardiomyopathy [3,39,40]. In our study, a decrease in myocardial T1 was associated with higher levels of NT-proBNP. Previous reports highlighted the possibility of low T1 findings in patients without the presence of LVH [41]. While other research groups initially demonstrated a correlation between NT-proBNP and the presence or fast increase of LGE, we could not confirm a significant interaction between NT-proBNP and the extent of LGE [42,43,44,45].

The prognostic value of NT-proBNP on disease severity and progression is also supported by the significant association of baseline NT-proBNP values with the FOS-MSSI in a linear regression model. Yet, LVH appears to be an important contributor as the linear regression model did not provide significant associations between NT-proBNP and the FOS-MSSI in non-hypertrophic individuals. It is important to note that the FOS-MSSI reflects overall disease severity, as it comprises the involvement of the multiple organ systems potentially affected by Fabry disease [13].

For the first time, our study reveals a strong correlation between the Fabry disease-specific laboratory marker LysoGb3 and NT-proBNP in a group of patients with LVH. In this context, the prognostic value of single laboratory biomarkers such as cardiac troponins, creatinine, cystatin C and albumin-to-creatinine ratio appears limited. However, a combination with LysoGb3 and NT-proBNP could overcome the individual diagnostical limitations and shed some light on various underlying pathophysiological aspects. Additionally, compared to protracted measurements of LysoGb3, NT-proBNP assessment is widely available and might constitute a quick possibility to evaluate the state of the disease. This finding emphasises the role of NT-proBNP measurement as part of the regular Fabry disease follow-ups [7,8,12]. Yet, the natural course of NT-proBNP is unknown, and the effects of specific treatment have only been examined by one study so far. The group of Nordin et al. discovered no changes in NT-proBNP levels after one year of enzyme replacement therapy [46]. Our data provide evidence for an initially non-significant trend towards higher NT-proBNP levels after 60 months, followed by a significant difference after 90 months in patients receiving specific treatment compared to those without. This course suggests that specific therapy may not fully address the underlying pathophysiology of Fabry disease or the consequences of glycosphingolipid accumulation. Additionally, the finding once more underlines the importance of this biomarker in longitudinal monitoring and might reflect factors such as progressive hypertrophy and aggravating cardiac involvement. Furthermore, our data suggest that patients with LVH and the need for specific therapy exhibit the highest NT-proBNP values as compared to those without these factors.

Past research focused on individual parameters derived from TTE or CMR and the consecutive NT-proBNP elevations. In contrast, our data further demonstrate the need for comprehensive cardiac follow-up, including cardiac imaging and laboratory biomarkers, as part of a multimodal approach. There are several findings to our work that constitute its novelty. We were able to highlight that NT-proBNP reflects left ventricular hypertrophy in Fabry patients. In the specific context of our cohort, markers of ventricular dilatation or dysfunction were found less determining. Moreover, the newly depicted association with low septal T1 and LysoGb3 could help to unveil pathophysiological aspects of Fabry disease. Our report on the longitudinal development of NT-proBNP under specific therapy might reflect continuous disease progression and is, to our knowledge, the only available documentation on long-term monitoring currently available in the literature. Finally, we aimed to shed some light on right ventricular involvement in Fabry disease and were able to depict findings reflecting those of the left ventricle.

However, this study faces limitations that warrant consideration. Firstly, the low sample size, attributed to the rarity of the disease, the analysis of patients suffering from different stages of Fabry disease, and a relevant number of patients presenting with a cardiac variant of Fabry disease restricts the generalizability of the findings and may affect the statistical power of the analysis. Secondly, the use of NT-proBNP as a biomarker poses challenges due to its lack of specificity for Fabry disease. Serum levels could be influenced by comorbidities such as chronic kidney disease, atrial fibrillation, and hypertension, potentially confounding the interpretation of the results. Thirdly, our data suggest no strong correlation between markers of left ventricular systolic function and NT-proBNP in Fabry patients. However, the low number of individuals with reduced LVEF may partially constitute this finding. Though this might be seen as a limitation, stemming from a real-world cohort, our data reflect the situation physicians are facing as well as the diagnostic value of NT-proBNP in this context. Lastly, the initiation of specific treatment was not standardised, leading to a variable duration of the therapy. Therefore, definitive conclusions regarding the therapeutic effects cannot easily be made. These limitations underscore the need for a cautious interpretation of the study findings and highlight areas for further investigation and refinement in future research endeavours.

## Figures and Tables

**Table 1:** Baseline characteristics (female vs. male patients).

	All Patients *n* = 38	Female *n* = 25	Male *n* = 13	*p*-Value
Age (years)	41 (27–50)	38 (27–46)	47 (36–57)	0.188
Time since diagnosis (months)	48 (9–96)	48.5 (8–93)	46 (11–96)	0.888
Left ventricular hypertrophy	18 (47.4%)	11 (44%)	7 (53.8%)	0.414
Specific therapy				
ERT	12 (31.6%)	6 (24%)	6 (46.2%)	0.163
Chaperone	3 (7.89%)	2 (8%)	1 (7.69%)	0.973
Time on therapy (months)	58 (20–99)	66.5 (17–108)	58 (20–89)	0.867
Laboratory markers				
NT-proBNP (pg/mL)	62.1 (30.1–166)	47.8 (28.7–146)	102 (42–344)	0.234
Creatinine (mg/dL)	0.79 (0.67–0.91)	0.73 (0.65–0.83)	0.92 (0.86–1.12)	**0.001**
eGFR (mL/min/1.73 m^2^)	105 (90.8–120)	105 (95.4–119)	104 (76–121)	0.724
ACR (mg/g)	205 (64–282)	140 (21–190)	269 (223–838)	**0.003**

ERT, enzyme replacement therapy; eGFR, estimated glomerular filtration rate (calculated using the CKD-EPI equation) [21]; ACR, albumin-to-creatinine ratio. A *p*-value of <0.05 was considered statistically significant (bold).

**Table 2:** Baseline symptoms, CMR and laboratory values among patients with or without left ventricular hypertrophy.

	All Patients *n* = 38	No LVH *n* = 20	LVH *n* = 18	*p*-Value
Age (years)	41 (27–50)	36 (23–47)	44 (34–50)	0.233
NYHA Class				
I	32 (84.2%)	17 (85%)	15 (83.3%)	
II	5 (13.2%)	3 (15%)	2 (11.1%)	
III	1 (2.6%)	0 (0%)	1 (5.6%)	
IV	0 (0%)	0 (0%)	0 (0%)	
Palpitations				
no	32 (84.2%)	15 (75%)	17 (94.4%)	
yes	6 (16.8)	5 (25%)	1 (5.6%)	
CCS Class				
No Angina	34 (89.5%)	20 (100%)	14 (77.8%)	
I	3 (7.9%)	0 (0%)	3 (16.7%)	
II	1 (2.6%)	0 (0%)	1 (5.5%)	
III	0 (0%)	0 (0%)	0 (0%)	
IV	0 (0%)	0 (0%)	0 (0%)	
CMR				
LVMI g/m^2^	70.6 (61–85)	62.4 (58.7–67.8)	85 (73.1–114)	**0.001**
LVEDV (mL)	134 ± 29.3	120 ± 22.7	148 ± 28.9	**0.003**
LVEF (%)	64.5 ± 7.73	63.4 ± 7.44	65.6 ± 8.08	0.413
Septal T1 (ms)	973 ± 85.8	1015 ± 48.5	946 ± 94.9	**0.039**
LGE (%)	0 (0–0)	0 (0–0)	0 (0–6.81)	0.577
Left atrial volume (mL)	61.9 (51.6–74.2)	63.2 (49.4–67.5)	61.5 (54.6–80)	0.413
RVEDV (mL)	133 ± 30	124 ± 25.3	143 ± 33	0.053
RVEF (%)	55.2 ± 6.78	53.6 ± 6.2	56.8 ± 7.2	0.15
Laboratory markers				
NT-proBNP (pg/mL)	62.1 (30.1–166)	44.5 (28.7–84.2)	144 (47.8–510)	**0.038**
Creatinine (mg/dL)	0.79 (0.67–0.91)	0.8 (0.69–0.93)	0.79 (0.65–0.91)	0.463
eGFR (mL/min/1.73 m^2^)	105 (90.8–120)	105 (89.1–119)	104.8 (95.95–121)	0.858
ACR (mg/g)	205 (64–282)	151 (8–239)	255 (205–1148)	0.055

LVH, left ventricular hypertrophy; CMR, cardiac magnetic resonance imaging; LVMI, left ventricular mass index; LVEDV, left ventricular end-diastolic volume; LVEF, left ventricular ejection fraction; RVEDV, right ventricular end-diastolic volume; RVEF, right ventricular ejection fraction; eGFR, estimated glomerular filtration rate (calculated using the CKD-EPI equation) [21]; ACR, albumin-to-creatinine ratio. A *p*-value of <0.05 was considered statistically significant (bold).

**Table 3:** Correlations between repetitive NT-proBNP measurements of the 38 patients and the respective echocardiographic parameters in the general cohort as well as in patients with or without left ventricular hypertrophy.

	All Measurements *n* = 72	Without LVH *n* = 25	With LVH *n* = 47
IVS thickness	**r = 0.641; *p* < 0.001**	r = 0.243; *p* = 0.276	**r = 0.678; *p* < 0.001**
Right atrial diameter	**r = 0.521; *p* < 0.001**	r = −0.379; *p* = 0.082	**r = 0.684; *p* < 0.001**
Aorta ascendens diameter	**r = 0.409; *p* < 0.001**	r = 0.146; *p* = 0.516	**r = 0.338; *p* = 0.031**
Right ventricular diameter	r = 0.009; *p* = 0.943	r = −0.413; *p* = 0.056	r = 0.076; *p* = 0.622
Left ventricular diameter	r = −0.104; *p* = 0.401	r = −0.288; *p* = 0.194	r = −0.06; *p* = 0.696
sPAP	**r = 0.76; *p* < 0.001**	r = 0.154; *p* = 0.651	**r = 0.865; *p* < 0.001**

IVS, interventricular septal thickness; sPAP, systolic pulmonary artery pressure. Bold indicates statistical significance.

**Table 4:** Correlations between repetitive NT-proBNP measurements of the 38 patients and the respective CMR parameters in the general cohort as well as in patients with or without left ventricular hypertrophy (LVH).

	All Measurements *n* = 80	Without LVH *n* = 34	With LVH *n* = 46
Left ventricle			
ED volume	r = −0.033; *p* = 0.771	r = −0.371; *p* = 0.031	r = 0.04; *p* = 0.795
ES volume	r = −0.144; *p* = 0.212	r = −0.21; *p* = 0.234	r = −0.067; *p* = 0.668
Stroke volume	r = 0.124; *p* = 0.282	r = −0.313; *p* = 0.072	r = 0.227; *p* = 0.143
Cardiac output	r = 0.031; *p* = 0.786	r = −0.23; *p* = 0.192	r = 0.059; *p* = 0.708
Ejection fraction	**r = 0.239; *p* = 0.037**	r = 0.034; *p* = 0.85	r = 0.192; *p* = 0.216
GLS	r = −0.008; *p* = 0.953	r = 0.254; *p* = 0.147	r = 0.029; *p* = 0.853
Myocardial mass	**r = 0.447; *p* < 0.001**	r = 0.098; *p* = 0.582	**r = 0.538; *p* < 0.001**
LVMI	**r = 0.494; *p* < 0.001**	r = 0.184; *p* = 0.298	**r = 0.549; *p* < 0.001**
Trabecular mass	**r = 0.407; *p* < 0.001**	r = 0.054; *p* = 0.766	**r = 0.526; *p* < 0.001**
Maximum wall thickness	**r = 0.594; *p* < 0.001**	r = −0.227; *p* = 0.502	**r = 0.717; *p* < 0.001**
Septal T1	**r = −0.302; *p* = 0.012**	r = −0.192; *p* = 0.338	r = −0.291; *p* = 0.065
LGE	r = 0.25; *p* = 0.068	r = −0.01; *p* = 0.959	r = 0.182; *p* = 0.374
Right ventricle			
ED volume	r = 0.164; *p* = 0.174	r = −0.025; *p* = 0.892	r = 0.111; *p* = 0.501
ES volume	r = −0.1; *p* = 0.414	r = −0.131; *p* = 0.489	r = −0.119; *p* = 0.469
Stroke volume	r = 0.234; *p* = 0.053	r = 0.036; *p* = 0.851	r = 0.197; *p* = 0.229
Cardiac output	r = 0.201; *p* = 0.097	r = 0.089; *p* = 0.64	r = 0.139; *p* = 0.397
Ejection fraction	**r = 0.299; *p* = 0.012**	r = 0.092; *p* = 0.624	r = 0.218; *p* = 0.182
Myocardial mass	**r = 0.367; *p* = 0.002**	r = 0.105; *p* = 0.575	**r = 0.470; *p* = 0.003**
RVMI	**r = 0.379; *p* = 0.001**	r = 0.142; *p* = 0.454	**r = 0.428; *p* = 0.007**
Trabecular mass	**r = 0.453; *p* < 0.001**	r = 0.093; *p* = 0.631	**r = 0.606; *p* < 0.001**

CMR, cardiac magnetic resonance imaging; ED, end-diastolic; ES, end-systolic; GLS, global longitudinal strain; LVMI, left ventricular mass index; LGE, late gadolinium enhancement; RVMI, right ventricular mass index. Bold indicates statistical significance.

## Data Availability

The raw data supporting the conclusions of this article will be made available by the authors upon reasonable request.

## Supplementary Materials

The following supporting information can be downloaded at https://www.mdpi.com/article/10.3390/jcm13195953/s1, Figure S1: NT-proBNP correlations and total cohort; Figure S2: Systolic and diastolic function and ventricular hypertrophy; Figure S3: NT-proBNP and markers of right ventricular hypertrophy, function and dilatation; Figure S4: Correlations of NT-proBNP values and imaging markers of systolic function and dilatation.
