# Supplementary material for: NT-proBNP Reflects Left Ventricular Hypertrophy Rather than Left Ventricular Dilatation or Systolic Dysfunction in Patients with Fabry Disease
Source: J Clin Med. 2024 Oct 7;13(19):5953. doi: 10.3390/jcm13195953 (PMC11478308; doi:10.3390/jcm13195953)
Supplement: Supplementary file 1 [file jcm-13-05953-s001.zip › jcm-3208030-supplementary.pdf]

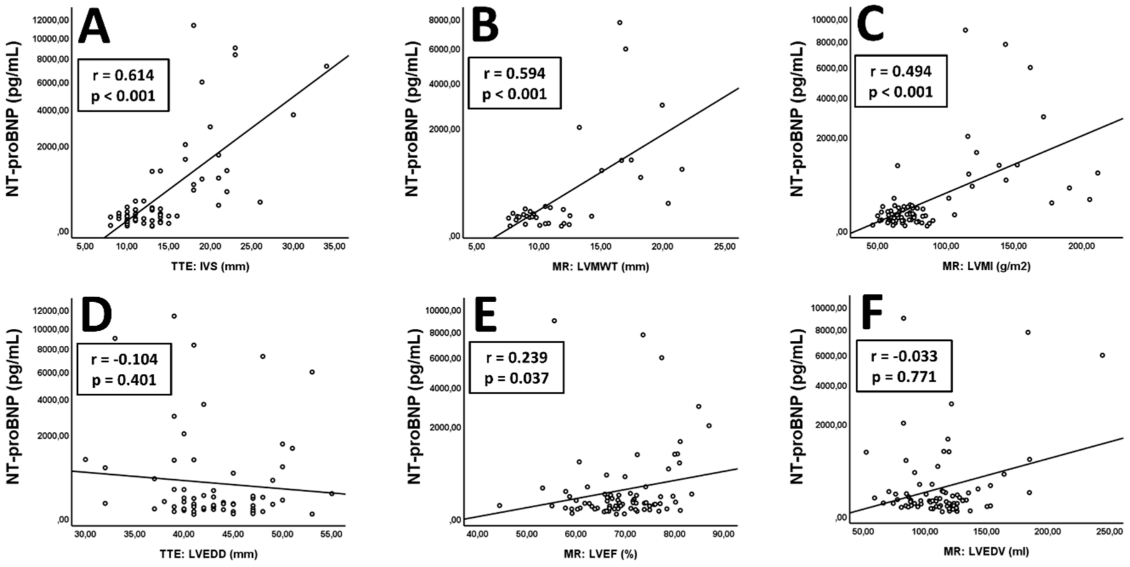

Suppl. Figure S1. Correlations of NT-proBNP values and imaging markers of left ventricular hypertrophy, function and dilatation in all included patients. The Y-axis is shown as a percentage function to highlight low NT-proBNP values. A p-value of < 0.05 was considered statistically significant.

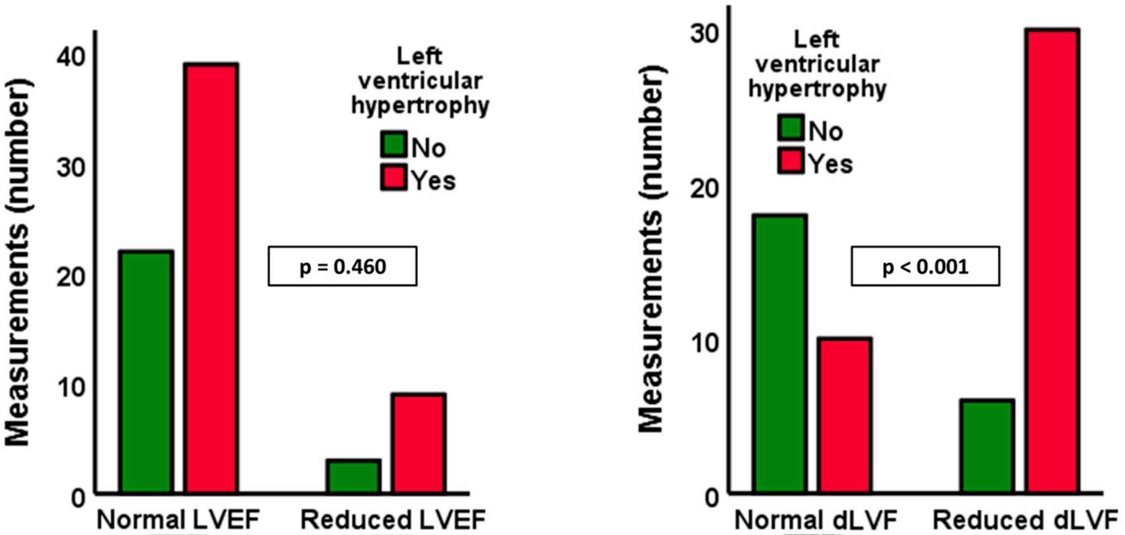

Suppl. Figure S2. Associations of systolic and diastolic function in conjunction with left ventricular hypertrophy. A p-value of < 0.05 was considered statistically significant.; TTE, transthoracic echocardiography; LVEF, left ventricular ejection fraction; dLVF, diastolic left ventricular function.

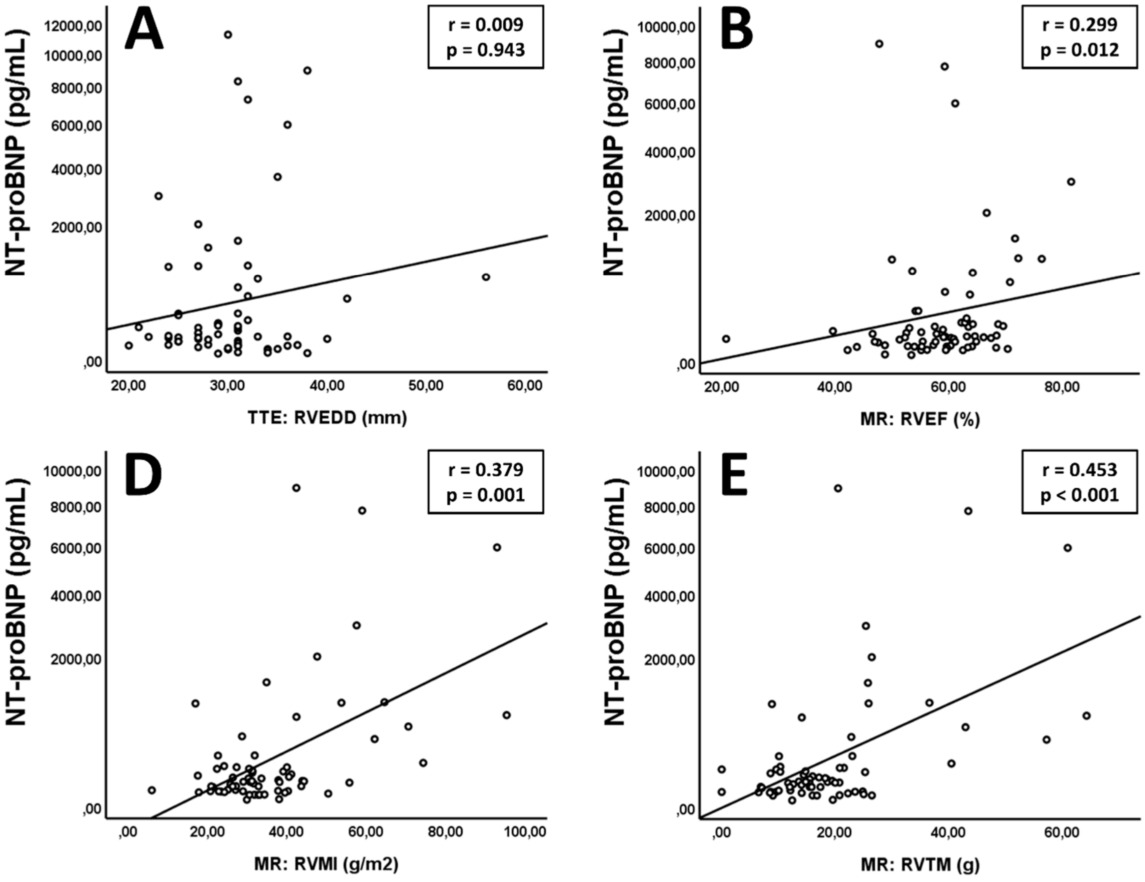

Suppl. Figure S3. Correlations of NT-proBNP values and imaging markers of right ventricular hypertrophy, function and dilatation in all included patients. The Y-axis is shown as a percentage function to highlight low NT-proBNP values. A p-value of < 0.05 was considered statistically significant.

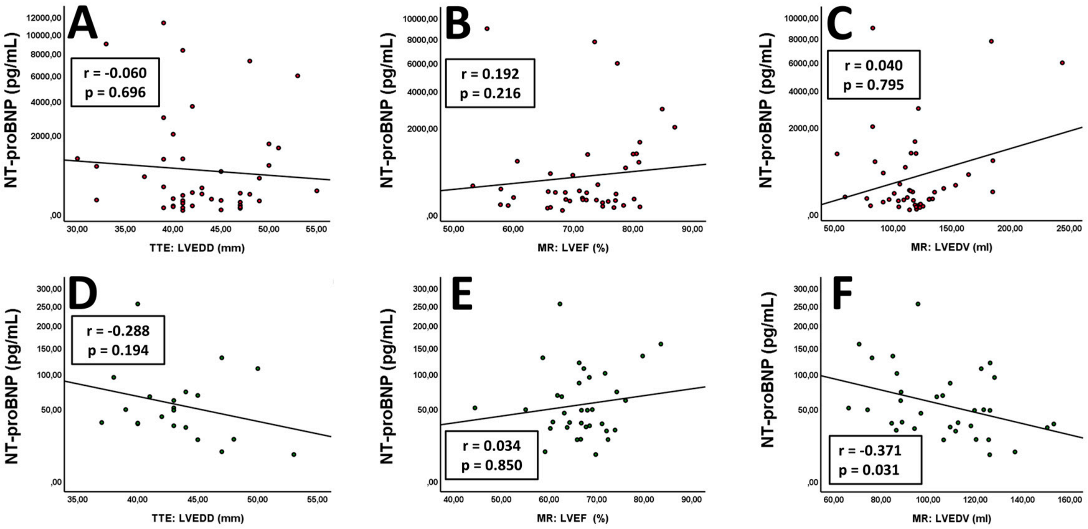

Suppl. Figure S4. Correlations of NT-proBNP values and imaging markers of systolic function and dilatation in patients with (Suppl. Figure 4A n = 45, 4B n = 44, 4C n = 46) or without (Suppl. Figure 4D n = 21, 4E n = 33, 4F n = 34) left ventricular hypertrophy. The Y-axis is shown as a percentage function to highlight low NT-proBNP values. A p-value of < 0.05 was considered statistically significant.
